# Healthcare seeking behavior of patients with influenza like illness: comparison of the summer and winter influenza epidemics

**DOI:** 10.1186/s12879-016-1821-7

**Published:** 2016-09-20

**Authors:** Huaiqing Meng, Qiuyan Liao, Lorna Kwai Ping Suen, Margaret O’Donoghue, Chit Ming Wong, Lin Yang

**Affiliations:** 1 School of Nursing, The Hong Kong Polytechnic University, Hung Hom, Hong Kong Special Administrative Region (HKSAR), China; 2 School of Public Health, The University of Hong Kong, Pokfulam, Hong Kong Special Administrative Region (HKSAR), China

**Keywords:** Healthcare seeking behavior, Influenza, Health services research, Seasonal variation

## Abstract

**Background:**

Influenza often causes winter and summer epidemics in subtropical regions, but few studies have investigated the difference in healthcare seeking behavior of patients with influenza-like illness (ILI) between these two epidemics.

**Methods:**

Household telephone surveys were conducted using random digit dialing in Hong Kong during July-August 2014 and March-April 2015. One adult from each household was interviewed for ILI symptoms and associated healthcare seeking behaviour of themselves and one child in the household (if any), during the preceding 30 days. Healthcare seeking behavior of respondents with self-reported ILI was compared between summer and winter influenza. Logistic regression was used to explore the factors associated with healthcare seeking behavior.

**Results:**

Among 516 and 539 adult respondents in the summer and winter surveys, 22.6 and 38.0 % reported ILI symptoms, and 40.9 and 46.8 % of them sought medical care, respectively. There was no significant difference in healthcare seeking behavior between the summer and winter epidemics, except a higher proportion of self-medication in summer in the adult respondents. Among 155 and 182 children reported by the adults in both surveys, the proportion of self-reported ILI was 32.9 and 40.1 % in the summer and winter surveys, respectively. Of these children, 47.1 and 56.2 % were brought for medical consultation in summer and winter, respectively. Women, adults with diabetes and those with symptoms of cough, shortness of breath, and runny nose were more likely to seek medical consultations for ILI symptoms. The factors associated with seeking medical consultations in children with ILI symptoms included being female, age under 10 years, and with symptoms of sore throat or vomiting. Those older than 60 years were less likely to self-medicate, whereas regular smokers and those with symptom of sore throat were more likely to do so.

**Conclusion:**

Healthcare seeking behavior of the general public was not significantly different between these two epidemics. However ILI was associated with increased healthcare utilization in both winter and summer epidemics in Hong Kong.

**Electronic supplementary material:**

The online version of this article (doi:10.1186/s12879-016-1821-7) contains supplementary material, which is available to authorized users.

## Background

Influenza is one of most contagious respiratory infectious diseases, and has nonspecific symptoms of fever, cough, sore throat, myalgia, headache and malaise. It is associated with higher morbidity and mortality, especially in the elderly and children [1]. Influenza epidemics occur predominately during winter months in temperate regions. However, in the tropics and subtropics, seasonal influenza viral transmission continues throughout the year [2]. In Hong Kong, influenza usually displays two peaks, one winter peak from February to March and another summer peak usually between June and July [3]. Healthcare seeking behavior of the general population could vary across winter and summer peaks due to perceived difference in severity and susceptibility to infections of diseases.

Most of the current surveillance systems for influenza are targeted on influenza-like illness (ILI) in clinical settings with the aim of detecting influenza epidemics and to issue timely alerts. In Hong Kong, ILI surveillance has been routinely conducted in all public General Outpatient Clinics and by a small sample of private practitioners [4]. However, a passive influenza surveillance system cannot capture patients with mild symptoms who do not consult practitioners nor understand the healthcare seeking behavior associated with ILI [5]. Previous studies have adopted the approach of household telephone surveys to capture symptomatic individuals, and have reported that ≥60 % of influenza infected individuals did not seek consultations but the proportion varied across regions [6, 7]. However, to our best knowledge, most of these studies were conducted during the 2009 H1N1 influenza pandemic and none compared the healthcare seeking behavior of ILI patients across the multiple influenza epidemics within the same season in the tropics and subtropics. This study aimed to compare healthcare seeking behaviors, such as seeking medical consultations in western practitioners or Traditional Chinese Medicine (TCM) and self-medication of ILI patients, between the summer and winter influenza epidemics in Hong Kong. The association of healthcare seeking behavior with demographic characteristics, underlying chronic conditions and respiratory symptoms were also investigated.

## Methods

### Telephone surveys

**Fig. 1:**
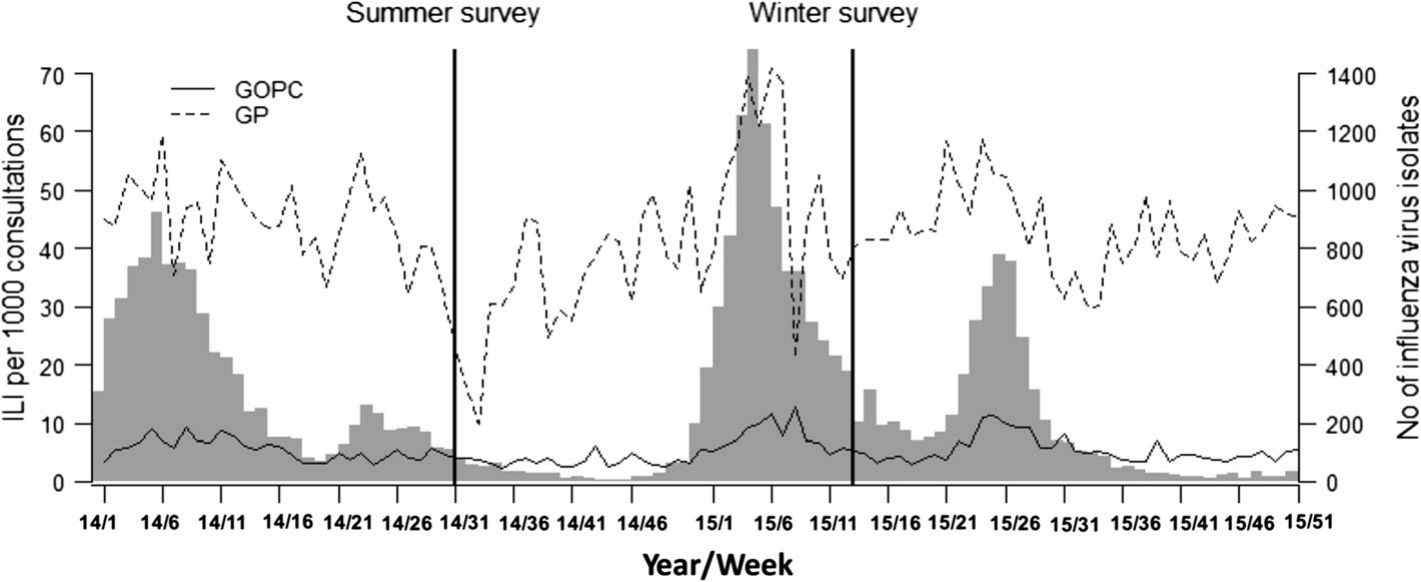
Weekly numbers of influenza isolates (*grey bar*) and ILI rates in Hong Kong (*lines*), 2014–2015. The data were downloaded from the Centre for Health Protection website http://www.chp.gov.hk/. The 2014 summer survey was conducted during 28 July-17 August 2014 and the 2015 winter survey during 23–27 March 2015. Abbreviations: GOPC, general outpatient clinics; GP, general practitioners

Two rounds of cross-sectional household telephone surveys were conducted by the professional interviewers from the Public Opinion Program of the University of Hong Kong on behalf of the research team. The survey periods selected were 28 July-17 August 2014 and 23–27 March 2015, when the summer and winter influenza peaks respectively had almost ended (Fig. 1). Both surveys asked the respondents to report influenza like symptoms and healthcare seeking behavior after symptoms in the preceding 30 days of the interview dates when influenza viruses were still active. These surveys were conducted separately, so households interviewed might be different.

Households were contacted by random digital dialling of landline telephone numbers in Hong Kong. One adult (age ≥18 years) within each household was selected by using the next-birthday method and was then invited for interview. The adult also answered the questions for one child aged <18 years within the same household, who was selected by the interviewers if there were two or more children. The interviews were conducted in Cantonese and each lasted around 5–10 min. The questionnaire included items on influenza-like symptoms and vaccination history, which were adopted from a series of telephone surveys previously conducted in Hong Kong [8]. Questions on healthcare utilization were slightly modified from the thematic household survey by the Census and statistics Department in 2010 [9]. The adult respondents were asked whether they (or their children, if any) visited public or private Western outpatient clinics, Tradition Chinese Medicine (TCM) practitioners, Accident and Emergency Department (A&E), and/or were hospitalized due to their previously reported symptoms. Self-medication with over-the-counter (OTC) drugs was also included in the questionnaire. Demographic data, education, occupation, housing type and lifestyle factors, such as smoking and exercise habits, were collected only for adult respondents. The adults were also asked to report any diagnosed pre-existing health conditions, including cardiovascular disease, cerebrovascular disease, renal disease, diabetes mellitus, chronic respiratory disease, liver disease, neurologic disease and hematologic disease. A pilot study was conducted on 11 July 2014 in 15 participants to test the validity and comprehensibility of the questionnaire, and those participants were not included in data analysis. Two extra questions about the pre-summer vaccine for Southern Hemisphere recommended strains were added to the questionnaire of winter surveys and the findings have been published elsewhere [10]. We estimated that the sample size of 500 subjects in each survey could have ≥80 % power to capture the expected ILI proportion of 10–50 % with a margin of error of 5 %.

### Data analysis

ILI in adults was defined as at least two of the signs or symptoms (fever ≥ 37.8 °C, cough, sore throat, headache, or myalgia) [11, 12]. As it has been reported that children seldom report headaches or myalgia, for this group ILI was defined as at least one respiratory symptom of fever ≥ 37.8 °C, cough or sore throat [13]. The probabilities of seeking medical care and self-medication were compared between the winter and summer epidemics by Chi-square test. Univariate and multivariate logistic regression models were used to explore the factors associated with medical care seeking behavior, including influenza-like symptoms, demographic characteristics, lifestyle factors, influenza vaccination history and chronic conditions. The forward method was adopted to select the variables to be included in the final multivariable model with a margin of *p* = 0.1. The goodness-of-fit of these models was assessed by the Hosmer-Lemeshow test. As a sensitivity analysis, we repeated the summer and winter comparison by using the ILI definition from European Center for Disease Control (ECDC): sudden onset of symptoms with at least one symptom of fever or feverish, malaise, head-ache, or myalgia, and at least one symptom of cough, sore throat, or shortness of breath [14]. SPSS version 22.0 was used for statistical analysis and the significance value was set at 0.05.

## Results

### Healthcare seeking behavior of adult and child respondents with ILI

**Fig. 2:**
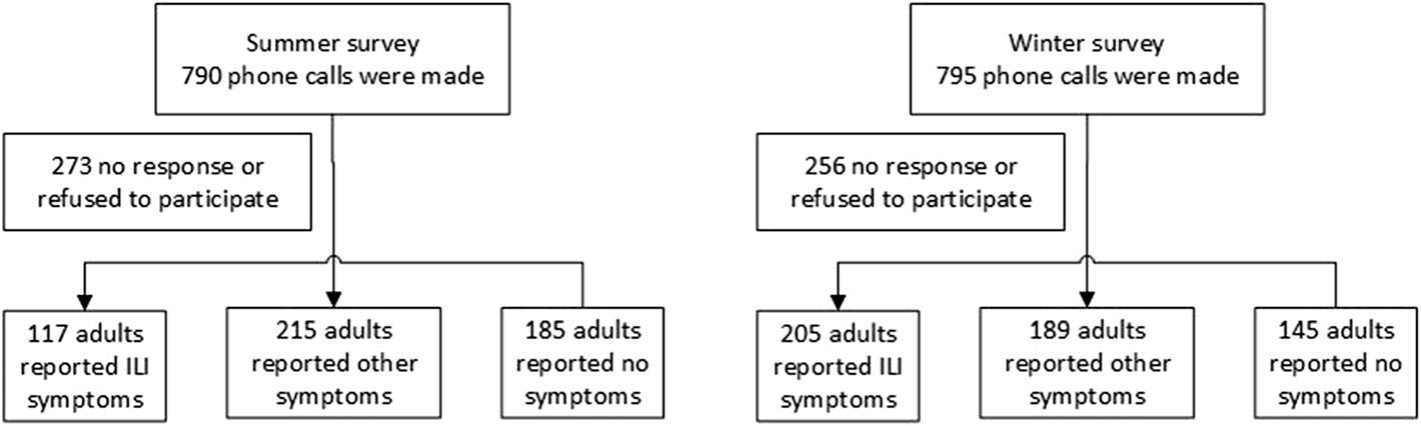
Flow chart of two telephone surveys

The flowchart of two telephone surveys is shown in Fig. 2. A total of 516 and 539 adults were successfully interviewed in the winter and summer surveys, and the response rates were 65.3 and 67.8 %, respectively. When compared to the general population of Hong Kong, the survey respondents were slightly older, had more female respondents, and attained a higher level of education (Table 1). Random Iterative Method (RIM) weighting was applied to adjust for the underrepresented population groups in raw data, by comparing the age and sex distribution of the year-end population of 2014 and 2015, and also education attainment in the 2011 population census [15]. The demographic characteristics of study participants in both surveys are shown in Additional file 1. The results of RIM-weighted data are presented hereafter in this paper.

**Table 1:** Comparison of demographic characteristics between adult respondents of two surveys and the general population of Hong Kong

	Summer survey respondents		Winter survey respondents		Hong Kong population^a^
	No.	%	No.	%	%
Age group (years)^b^	505		535		
18–59	314	60.8	353	65.5	75.4
> 60	191	37.8	182	33.8	24.6
Female	302	58.5	340	63.1	54.7
Education^c^	512		533		
Primary or below	99	19.3	91	16.9	23.4
Secondary	233	45.5	263	48.8	50.5
Tertiary or above	180	35.1	179	33.2	26.1

^a^Percentage of age group was calculated based on the population of mid–2014 and the education data from the 2011 population census

^b^There were eleven and four respondents who refused to give age in summer and winter surveys

^c^Four and six respondents did not answer education attainment in the summer and winter surveys, respectively

There were 117 (22.6 %) and 205 (38 %) adults who reported ILI symptoms in the summer and winter surveys, respectively. Among the adults with self-reported ILI, 40.9 and 46.8 % sought medical care in the summer and winter peaks (Additional file 2). There was no significant difference between summer and winter in the probability of medical care seeking behavior among the ILI adults, except the proportion of adults who chose self-medication (44.4 % in summer vs. 29.6 % in winter). There was no significant difference between the ILI adults who sought medical care in summer and winter, in terms of their age, gender, marital status, occupation, family income and living districts, underling chronic diseases except for those with pre-existing cardiovascular diseases. Some occupations (manager, administrator, and professionals), and those with underlying chronic respiratory disease were more likely to take self-medication in summer (Additional file 2).

Of 155 and 182 adults reporting for children in their households in the summer and winter surveys, 51 (32.9 %) and 73 (40.1 %) of children had ILI in the 2014 summer and 2015 winter peaks. 47.1 and 56.2 % of children with influenza-like symptoms had medical consultations in summer and winter, respectively (Table 2). A larger proportion of girls sought medical consultations in the summer peak (Table 2).

**Table 2:** Demographic characteristics of children with influenza-like illness and those who also sought medical care

Demographic characteristics	2014 summer			2015 winter			*p*-value^a^
	Reported ILI	Sought medical care		Reported ILI	Sought medical care		
	No.	No.	%	No.	No.	%	
No.	51	24	47.1	73	41	56.2	0.318
Age group (years)							0.638
0–5	11	5	45.5	16	12	75.0	
5–10	16	9	56.3	17	13	76.5	
10–18	24	10	40.0	39	15	39.5	
Sex							**0.038**
Male	25	6	24.0	38	21	55.3	
Female	26	18	69.2	35	20	57.1	

ILI, influenza like illness. Weighted data are presented in this table

^a^ *p*-value of Chi-square tests among influenza like illness cases who sought medical care in summer and winter surveys

### Comparison of medical care seeking behavior between the summer and winter epidemics

There was no significant difference between summer and winter in the probability of different patterns of medical care seeking behavior among the ILI adults (Table 3). Most of adults with ILI visited private western clinics (57.0 % in summer and 70.1 % in winter), and few chose to visit the public TCM outpatient clinics (0 and 2.1 %). Only 8.6 % of all adult respondents with ILI in summer and 3.2 % in winter reported hospital admission episodes associated with ILI. Similar to adults, most children visited the western private clinics. Children with ILI were more likely to be brought to private western clinics in the winter peak (80.5 % vs. 50.0 %), but less likely to public western clinics (4.9 % vs. 33.3 %) and to be hospitalized into private hospitals (0.0 % vs. 16.7 %) than in the summer peak (Table 3). Of adults with self-reported influenza-like symptoms, 21.3 % in summer and 25.7 % in winter reported multiple visits to one type or different types of medical care providers. The corresponding proportions in children with reported influenza-like symptoms were 24.1 % in summer and 32.6 % in winter. The sensitivity analysis using the ECDC definition identified slightly fewer ILI cases, but the results were very similar to the main analysis (Additional file 3).

**Table 3:** Medical care seeking behavior of adult and children with influenza like illness

	2014 Summer		2015 Winter		*p*-value^a^
	No.	%	No.	%	
Adults					
Sought medical care^b^	47		94		
Private					
A&E	6	12.8	15	16.0	0.432
Western clinic	27	57.4	66	70.2	0.087
TCM	15	31.9	23	24.5	0.660
Hospitalization	2	4.3	1	1.1	0.169
Public					
A&E	3	6.4	5	5.3	1.000
Western clinic	15	31.9	17	18.1	0.197
TCM	0	0.0	2	2.1	0.533
Hospitalization	2	4.3	2	2.1	1.000
Children					
Sought medical care ^b^	24		41		
Private					
A&E	6	25.0	10	24.4	0.752
Western clinic	12	50.0	33	80.5	**0.014**
TCM	3	12.5	7	17.1	0.523
Hospitalization	4	16.7	0	0.0	**0.008**
Public					
A&E	0	0.0	4	9.8	0.143
Western clinics	8	33.3	2	4.9	**0.016**
TCM	0	0.0	0	0.0	NA
Hospitalization	0	0.0	0	0.0	NA

*A&E* accident and emergency department, *TCM* traditional Chinese medicine

^a^ *p*-value of Chi-square tests

^b^Different types or multiple episodes of medical care events reported by one participant were counted as only one episode

### Symptoms and factors associated with medical consultations and self-medication

92.4 % of ILI adults who sought medical care reported three out of a total eleven symptoms, and 94.6 % of children who sought medical care reported at least two out of a total eight symptoms. In adults and children with ILI, there was a significant difference in number of symptoms between those who sought medical care and those who did not (Additional file 4).

The factors associated with medical care seeking behavior in adults include some influenza-like symptoms (fever, chills, headache, cough, shortness of breath, runny or stuffy nose, and sore throat), sex, age group, education, influenza vaccination and chronic diseases (cardiovascular, chronic respiratory, liver diseases and diabetes). But only the variables of cough, shortness of breath, runny or stuffy nose, sex, education and diabetes remained significant in the multivariate model. Women, adults with diabetes, or those who reported symptoms of cough, shortness of breath, runny or stuffy nose were more likely to seek consultations (Table 4). Those older than 60 years were less likely to take self-medication, but the current regular smokers and those with influenza-like symptoms of sore throat were more likely to do so (Table 5). In children, Girls, children under 10 years old, and those who had symptoms of sore throat or vomiting were more likely to see doctors (Table 6).

**Table 4:** Factors associated with medical care seeking behavior of the adults with self-reported influenza like illness

Factors	Univariate				Multivariate			
	Raw OR	95 % CI		*p*-value	Adjusted OR	95 % CI		*p*-value
Sex								
Male	ref				ref			
Female	1.49	0.95	2.35	0.085	2.49	1.44	4.32	**0.001**
Age group (years)								
18–39	ref							
40–59	1.46	0.88	2.41	0.144				
≥ 60	2.24	1.20	4.16	**0.011**				
Education				**0.002**				
Tertiary or above	ref							
Secondary	0.76	0.44	1.30	0.312				
Primary or below	2.20	1.14	4.23	**0.019**				
Symptoms								
Fever	3.22	1.24	8.40	**0.017**				
Chills	1.01	0.52	1.97	0.967				
Headache	0.91	0.58	1.43	0.687				
Myalgia	0.57	0.34	0.96	**0.033**				
Cough	1.96	1.23	3.11	**0.004**	2.32	1.34	4.05	**0.003**
Shortness of breath	2.68	1.40	5.13	**0.003**	3.11	1.47	6.61	**0.003**
Dizziness	1.25	0.77	2.04	0.363				
Runny or stuffy nose	2.02	1.27	3.20	**0.003**	1.92	1.14	3.24	**0.014**
Sore throat	1.52	0.97	2.39	0.069				
Diarrhea	0.76	0.45	1.28	0.298				
Low back pain	1.20	0.77	1.87	0.427				
Chronic disease								
Cardiovascular diseases	3.94	1.38	11.22	**0.010**				
Chronic respiratory	2.053	1.01	4.19	**0.048**				
Liver	0.24	0.06	1.00	**0.050**				
Diabetes	2.23	0.96	5.13	0.061	2.51	1.01	6.22	**0.047**
Flu vaccination								
2014/2015 season	2.07	1.08	3.95	**0.028**				
Every year in the past 3 years	2.12	1.06	4.21	**0.033**				

Weighted data of adults with self-reported influenza like illness in two surveys were combined in this analysis. Variables in multivariate models were selected by using the forward stepwise method with the cut-off point of *p*-value =0.1

**Table 5:** Factors associated with self-medication of the adults with self-reported influenza like illness

Factors	Univariate				Multivariate			
	Raw OR	95 % CI		*p*-value	Adjusted OR	95 % CI		*p*-value
Age group (years)				**0.001**				**<0.001**
18–39	ref				ref			
40–59	1.30	0.79	2.14	0.311	1.38	0.80	2.38	0.279
> =60	0.27	0.12	0.60	**0.001**	0.26	0.11	0.60	**0.002**
Education				0.064				
Primary or below	ref							
Secondary	2.14	1.12	4.07	**0.021**				
vTertiary or above	2.00	0.98	4.10	0.058				
Family income (Hong Kong dollar)				**0.014**				
< 10000	ref							
10000–20000	2.04	1.05	3.98	0.**036**				
20000–30000	2.77	1.34	5.75	**0.006**				
30000–40000	1.06	0.38	2.90	0.918				
> 40000	2.69	1.35	5.37	**0.005**				
Smoking habit				**0.022**				**0.019**
Never smoker	ref				ref			
Ex-smoker	0.41	0.19	0.93	**0.033**	0.55	0.23	1.31	0.178
Occasional smoker	0.31	0.08	1.32	0.113	0.30	0.07	1.30	0.107
Regular smoker	1.70	0.80	3.63	0.169	2.65	1.11	6.34	**0.029**
Symptoms								
Runny or stuffy nose	1.59	0.99	2.56	0.057				
Sore throat	1.55	0.97	2.49	0.068	1.75	1.04	2.97	**0.036**
Flu vaccination								
2014/2015 season	0.27	0.11	0.65	**0.003**				

Weighted data were used in this analysis. Variables in multivariate models were selected by using the forward stepwise method with the cut-off point of *p*-value =0.1

**Table 6:** Factors associated with medical care seeking of children with influenza like illness

Factors	Univariate				Multivariate			
	OR	95 % CI		*P*-value	Adjusted OR	95 % CI		*P*-value
Age group (years)								
10–18	ref				ref			
5–10	3.10	1.28	7.51	**0.012**	4.23	1.48	12.04	**0.007**
0–5	2.78	1.09	7.05	**0.032**	3.57	1.21	10.47	**0.021**
Sex								
Boy	ref				ref			
Girl	2.18	1.06	4.47	**0.034**	3.75	1.57	8.96	**0.003**
Symptoms								
Fever	3.46	1.56	7.67	**0.002**				
Sleepy	0.71	0.21	2.38	0.578				
Sore throat	2.63	1.27	5.44	**0.009**	2.43	1.03	5.70	**0.042**
Runny nose	1.30	0.60	2.80	0.509				
Cough	0.77	0.33	1.77	0.531				
Diarrhoea	1.49	0.61	3.62	0.382				
Vomiting	12.05	1.72	84.61	**0.012**	14.29	1.84	110.74	**0.011**
Dehydration	4.66	0.14	160.05	0.394				

Weighted data were used in this analysis. Variables in multivariate models were selected by using the forward stepwise method with the cut-off point of *p*-value =0.1

## Discussion

Our study is the first to compare individual healthcare seeking behavior after influenza like illness between two seasonal influenza epidemics in subtropical regions. Both adults and children were more likely to seek medical care in winter than in summer, although the difference was not significant. The only significant difference between summer and winter was the self-medication of ILI adults, with a higher probability in the summer peak (44.4 % in summer vs. 29.6 % in winter). Our previous studies have demonstrated that influenza tended to be milder and caused fewer deaths in the summer epidemics than in winter, regardless of predominant influenza strains [16, 17]. However, we did not observe a higher consultation rate during the large outbreak of influenza A (H3N2) viruses in the winter of 2015 (Fig. 1). This suggests that there are many other factors associated with medical consultations but unmeasured in our study. People tend to stay more vigilant against influenza in winter as the association of cold weather with respiratory viruses has long been recognized. Also in winter there is usually a wide media coverage of influenza outbreaks in schools, kindergartens and hospitals. Insurance coverage, less work load and medical certificate requirement for sick leave could increase the chance of seeking medical care, but they are less likely to change healthcare seeking behavior in different seasons.

In our study, the self-reported rate of ILI in the survey was 22.6–38.0 % for adults and 32.9–40.1 % for children, which was slightly higher than the telephone survey conducted in the US during the 2009 H1N1 pandemic (8.1 % of adults and 28.4 % of children), and another survey in 2010–2011 (8.1 % of adults and 34.0 % of children) [18, 19]. The reason for different ILI rates between the US studies and ours could be due to the fact that the symptom of fever was required to define ILI cases in the US studies but not in ours. The ILI definition of measured fever of ≥38 °C and cough has been adopted in most surveillance systems because body temperature is routinely measured in the clinical settings. However, for community settings where people may not have access to thermometers or seldom take body temperature, alternative definitions might be needed. In fact, fever of ≥38 °C was reported only in 1.5 % of our adult respondents in the summer survey and 2.9 % in the winter one. The discrepancy between our study and the US studies could also be due to the longer durations of two US surveys (7 and 4 months), which could have decreased the incidence rate due to inclusion of the nonepidemic weeks.

This study also revealed that more Hong Kong people chose private western clinics for medical care consultation. This is in line with the medical care behavior of the general population in Hong Kong. A recent Hong Kong population survey reported that 56.8 % of those who had consulted a doctor in the month before interview chose a private practitioner of western medicine [20]. The results of our study may provide important evidence to facilitate related policymaking and to allocate medical resources in a reasonable way.

Our findings on factors associated with different healthcare seeking behaviors were largely consistent with the previous studies. We found that 41–47 % of adults with ILI and 47–56 % of children sought medical care. These results are similar to the US seasonal influenza survey (45 % of adults and 57 % of children) [19], but slightly lower than those observed in France and Laos during the 2009 influenza pandemic (70 and 71 % for all ages) [7, 21]. We also found that adults with low education were more likely to seek medical care. This finding is in line with those of previous studies in both developed and developing countries/regions [22]. Similar to previous studies, chronic cardiovascular and respiratory diseases were associated with a higher rate of medical care seeking in ILI adults [18, 19], probably because these conditions have been well recognized as risk factors for developing severe complications after influenza infection. Consistent with previous studies [19], women or girls with influenza-like symptoms were more likely to seek a medical consultation when they had influenza like symptoms, as compared to men or boys. Having diabetes was also found associated with a higher probability of seeking consultations. It has been widely demonstrated that diabetes increases the risk of influenza infections and thereby the likelihood of medical consultation, hospitalization, ICU admission, and mortality attributable to influenza [23]. Similar to previous studies in France and US [7, 19], children <10 years had a significantly higher consultation rate than the other age groups. However, the results need to be interpreted with caution in our study, because of the relatively small number of subjects who reported for their children. The influenza-like symptoms associated with a higher probability of seeking medical care in adults were cough, shortness of breath, runny or stuffy nose. Previous studies have reported that adults with shortness of breath or cough had higher consultation rate to general physician [24]. Our results in children also echo the findings of Hsieh et al. by showing that the symptoms of sore throat or vomiting were possibly associated with medical consultations [25]. Due to the limited time in conducting telephone interviews, there are still many factors that remain unexplored in our study, such as traveling history, lifestyle factors and vaccination status. These factors could be considered in future studies.

Previous studies have shown that vaccinated people were prone to believe that they were more likely to catch influenza and that influenza could be a severe disease [26]. In this study, receiving influenza vaccination was associated with a higher probability of seeking medical care in adults and children with ILI in univariate analysis, but this was no longer significant after including other variables in multivariate analysis in adults. In children, the goodness-of-fit of logistic regression model according to the Hosmer-Lemeshow test could not be satisfied if the variable of influenza vaccination was included. This might be due to the small number of children with ILI in the survey.

There are a few limitations in this study. First, recall bias might have occurred, as the respondents were asked to recall symptoms during the preceding 30 days of interview dates. Second, we found that the interviewed subjects tended to be older, with more women and a higher level of education than the general population of Hong Kong, suggesting the existence of selection bias. Hence we used the RIM approach to increase the representativeness of our data. We did not include mobile phone numbers in our subject selection pool due to a high number of tourists in Hong Kong and the high penetration of landline telephones in the households of Hong Kong (>98 %) [8]. Third, this study was a community based household survey; hence those who are institutionalized were not being interviewed and this may affect the generalization of our findings. Last but not lease, this study was based on self-reported symptoms to define ILI cases, whereas many could have been infected by other pathogens. Only two surveys were conducted during the 2014 summer peak and 2015 winter peak, which were dominated by the same influenza strains of A/Texas/50/2012 (H3N2) [5]. This may not be able to provide the full picture of healthcare seeking behavior due to the short study period.

## Conclusions

Healthcare seeking behavior of the general public was not significantly different between these two epidemics, however, influenza was associated with increased healthcare utilization in both winter and summer epidemics in Hong Kong. This study also provides useful information on ILI incidence in the community, which can be integrated into a comprehensive assessment of the healthcare needs and economic burden of influenza in the entire Hong Kong population. Moreover, our findings highlight the need for increasing influenza surveillance sites in sentinel private clinics, which could provide more reliable and real-time surveillance information. In future studies, influenza vaccination rate and preventive measures among individuals with ILI, non-ILI and asymptomatic cases in the community could be assessed in order to efficiently establish and effectively implement targeted, practical and appropriate strategies to control influenza infections.

## Additional files

Additional file 1: Comparison of the demographic characteristics of all the respondents with and without weighting by the random iterative method. (XLSX 15 kb) Additional file 2: Self-reported influenza like illness, medical care seeking and self-medication in adults by selected demographics. (XLSX 16 kb) Additional file 3: Sensitivity analysis on healthcare seeking behavior of the adult respondents with influenza like illness using the alternative case definitions. (PDF 60 kb) Additional file 4: Symptoms and signs of individuals with influenza like illness who sought medical care. (PDF 50 kb)

## Abbreviations

A & E: Accident and emergency department
GP: General physician
ILI: Influenza-like illness
OTC: Over-the-counter
RIM: Random iterative method
SPSS: Statistic package for social science
TCM: Traditional Chinese medicine
USCDC: US centers for disease control and prevention
