# Supplementary material for: Healthcare seeking behavior of patients with influenza like illness: comparison of the summer and winter influenza epidemics
Source: BMC Infect Dis. 2016 Sep 20;16:499. doi: 10.1186/s12879-016-1821-7 (PMC5029067; doi:10.1186/s12879-016-1821-7)
Supplement: Additional file 3: — Sensitivity analysis on healthcare seeking behavior of the adult respondents with influenza like illness using the alternative case definitions. (PDF 60 kb) [file 12879_2016_1821_MOESM3_ESM.pdf]

Additional file 3. Sensitivity analysis on healthcare seeking behavior of the adult respondents with influenza like illness using the alternative case definitions.

	2014 Summer		2015 Winter		
	No.	%	No.	%	*p*-value [a]
**ILI**	91		143		
**Self-medication**	41	45.6 [c]	42	29.2 [c]	**0.011**
**Sought medical care** [b]	40	44.0 [c]	67	46.9 [c]	0.665
**Private**					
A&E	6	15.0	10	14.9	0.911
Western clinic	25	62.5	45	67.2	0.515
TCM	13	32.5	16	23.9	0.466
Hospitalization	2	5.0	1	1.5	0.247
**Public**					
A&E	4	10.0	3	4.5	0.434
Western clinic	11	27.5	16	23.9	0.813
TCM	0	0.0	1	1.5	1.000
Hospitalization	2	5.0	2	3.0	1.000

A&E: accident and emergency department; TCM: traditional Chinese medicine.
[a] *p*-value of Chi-square tests
[b] Different types or multiple episodes of medical care events reported by one participant were counted as only one episode.
[c] Percentage of healthcare seeking behavior in total number of ILI.
