# Supplementary material for: Healthcare seeking behavior of patients with influenza like illness: comparison of the summer and winter influenza epidemics
Source: BMC Infect Dis. 2016 Sep 20;16:499. doi: 10.1186/s12879-016-1821-7 (PMC5029067; doi:10.1186/s12879-016-1821-7)
Supplement: Additional file 4: — Symptoms and signs of individuals with influenza like illness who sought medical care. (PDF 50 kb) [file 12879_2016_1821_MOESM4_ESM.pdf]

Additional file 4. Symptoms and signs of individuals with influenza like illness who sought medical care.

**Symptoms**	**Medical care seeking**	
	**n**	**%**
**Adults**		
Fever	15	71.4
Chills	18	45.0
Headache	79	43.6
Myalgia	100	41.3
Cough	96	51.1
Shortness of breath	30	65.2
Dizziness of breath	45	48.9
Runny or stuff nose	96	51.3
Sore throat	89	48.9
Diarrhoea	31	39.7
Low back pain	67	47.2
**Children**		
Fever	31	72.1
Sleepy	5	41.7
Sore throat	41	64.1
Runny nose	47	54.0
Cough	48	50.5
Diarrhoea	15	60.0
Vomit	12	92.3
Dehydration	2	100.0
